# Impact of reduced antibiotic treatment duration on antimicrobial resistance in critically ill patients in the randomized controlled SAPS-trial

**DOI:** 10.3389/fmed.2023.1080007

**Published:** 2023-02-02

**Authors:** Arezoo Shajiei, Matthijs S. Berends, Christian F. Luz, Jos A. van Oers, Hermie J. M. Harmsen, Piet Vos, Rob Klont, Bert G. Loef, Auke C. Reidinga, Laura Bormans-Russell, Kitty Linsen, Tom Dormans, Martine Otten, Akke van der Bij, Albertus Beishuizen, Dylan W. de Lange, Evelien de Jong, Maarten W. Nijsten

**Affiliations:** ^1^Department of Critical Care, University Medical Center Groningen, Groningen, Netherlands; ^2^Department of Medical Microbiology, University Medical Center Groningen, Groningen, Netherlands; ^3^Department of Medical Epidemiology, Certe Foundation, Groningen, Netherlands; ^4^Department of Intensive Care, Elisabeth-Tweesteden Ziekenhuis, Tilburg, Netherlands; ^5^Laboratorium Microbiologie Twente Achterhoek, Hengelo, Netherlands; ^6^Department of Intensive Care, Martini Hospital Groningen, Groningen, Netherlands; ^7^Department of Intensive Care, Zuyderland Medical Center, Heerlen, Netherlands; ^8^Department of Intensive Care, Diakonessenhuis Utrecht, Utrecht, Netherlands; ^9^Department of Microbiology and Immunology, Diakonessenhuis Utrecht, Utrecht, Netherlands; ^10^Intensive Care Center, Medisch Spectrum Twente, Enschede, Netherlands; ^11^Department of Intensive Care, University Medical Center Utrecht, Utrecht, Netherlands; ^12^Department of Intensive Care, Beverwijk Hospital, Beverwijk, Netherlands; ^13^Department of Intensive Care, Amsterdam University Medical Center, Amsterdam, Netherlands

**Keywords:** antibiotics, antimicrobial resistance, procalcitonin, treatment duration, culture, randomized trial

## Abstract

**Background:**

In the previously reported SAPS trial (https://clinicaltrials.gov/ct2/show/NCT01139489), procalcitonin-guidance safely reduced the duration of antibiotic treatment in critically ill patients. We assessed the impact of shorter antibiotic treatment on antimicrobial resistance development in SAPS patients.

**Materials and methods:**

Cultures were assessed for the presence of multi-drug resistant (MDR) or highly resistant organisms (HRMO) and compared between PCT-guided and control patients. Baseline isolates from 30 days before to 5 days after randomization were compared with those from 5 to 30 days post-randomization. The primary endpoint was the incidence of new MDR/HRMO positive patients.

**Results:**

In total, 8,113 cultures with 96,515 antibiotic test results were evaluated for 439 and 482 patients randomized to the PCT and control groups, respectively. Disease severity at admission was similar for both groups. Median (IQR) durations of the first course of antibiotics were 6 days (4–10) and 7 days (5–11), respectively (*p* = 0.0001). Antibiotic-free days were 7 days (IQR 0–14) and 6 days (0–13; *p* = 0.05). Of all isolates assessed, 13% were MDR/HRMO positive and at baseline 186 (20%) patients were MDR/HMRO-positive. The incidence of new MDR/HRMO was 39 (8.9%) and 45 (9.3%) in PCT and control patients, respectively (*p* = 0.82). The time courses for MDR/HRMO development were also similar for both groups (*p* = 0.33).

**Conclusions:**

In the 921 randomized patients studied, the small but statistically significant reduction in antibiotic treatment in the PCT-group did not translate into a detectable change in antimicrobial resistance. Studies with larger differences in antibiotic treatment duration, larger study populations or populations with higher MDR/HRMO incidences might detect such differences.

## Introduction

Antibiotic treatment should be optimized in terms of its spectrum and duration to maximize patient outcome whilst minimizing the development potential antimicrobial resistance (AMR) and other side effects (1–3). Efforts to limit AMR in the intensive care unit (ICU) are of particular importance (4–7). In the stop antibiotics on guidance of PCT study (SAPS) (8), that randomized 1,546 ICU patients to PCT-guidance or standard-of-care, we observed a safe reduction of antibiotic treatment duration (ABTD) to a median of 5 days compared to 7 days with standard-of-care.

Interventions that lead to a reduced overall antibiotic consumption would be expected to lead to a reduction in AMR. Some randomized studies outside the ICU indeed observed reduced AMR (9), but for ICU studies that randomized up to 604 patients no significant impact on AMR was seen (10–13). Thus, the effect of reduced ABTD on AMR might be detectable in the larger cohort of ICU patients that was used in the SAPS trial.

The aim of the current study was to assess if the reduced ABTD achieved in the PCT arm of the SAPS study had an impact on the development of AMR.

## Methods

The SAPS trial design (14) and its findings (8) have been published previously. This study was approved for all centers by the Ethics Committee of the Amsterdam University Medical Center and is in compliance with the Helsinki Declaration. SAPS was performed in 15 hospitals in the Netherlands between 2009 and 2013 (https://clinicaltrials.gov/ct2/show/NCT01139489). Adult patients admitted to the ICU and treated for presumed bacterial infection, were randomized after informed consent. PCT was measured daily in the intervention arm. When PCT showed an absolute level of ≤0.5 μg/L or a relative decrease to ≤20% of the baseline level, a non-binding advice was given to consider to discontinue antibiotic treatment.

For the current substudy, seven of the participating institutions were able to provide the required complete culture and resistance data from their hospital information systems. Microbiological data (i.e., type of culture and microorganisms cultured) from specimens from all sources were obtained for −30 to +30 days relative to randomization and prospectively recorded in the case record form during the trial. All reported isolates were then combined into a single database with source of the material, cultured microorganism and resistances recorded in a standardized manner. Resistances were obtained after conclusion of the SAPS trial and were classified as sensitive, intermediate and resistant, following automated standardized antimicrobial susceptibility testing. To compare the impact of reduced ABTD, we compared baseline resistance data with data obtained after randomization. Since most cultures were obtained directly after ICU-admission and randomization, and because resistance typically does not become manifest within a few days (15), we chose as baseline period the interval from −30 to +5 days relative to randomization. This baseline period was compared with the subsequent period, i.e., +5 to +30 days. To determine the incidence of new MDR/HRMO positive patients, microorganisms were classified as MDR based on an international definition from 2012 (16). The HRMO-classification was based on Dutch guidelines^1^,^2^ as also further detailed in the Supplementary material. Both classifications were dichotomized to negative and positive, where positive denotes any form of multidrug resistance. Since our key data concerned AMR, which is generally considered unsuitable for imputation, no techniques were used make data more complete. All available cultures were examined for multi-drug resistant (MDR) or highly drug resistant (HRMO) organisms and the change in MDR/HRMO status was the primary endpoint.

The chi-square, Mann–Whitney *U* and Student's *t*-tests were used for group comparisons with two-sided *p*-values. The actuarial cumulative percentages for the first occurrence of an MDR/HRMO isolate were compared with the Kaplan–Meier method for the PCT and control groups with the log-rank test.

## Results

We evaluated 921 (60%) of the original 1,546 patients that were included in the SAPS trial. The numbers of patients randomized to PCT-guidance and standard of care were 439 and 482, respectively. The baseline characteristics of these groups are shown in Table 1. Severity of illness and other baseline indicators were similar. The most observed presumed infection was community acquired pneumonia. The median (IQR) durations of the first course of antibiotics were 6 days (4–10) and 7 days (5–11), respectively (*p* = 0.0001) with a difference of 1.03 days between the means (Table 2). ICU and hospital length of stay and 28 day mortality were similar.

**Table 1:** Baseline characteristics.

	**PCT group (*n* = 439)**	**Standard-of-care group (*n* = 482)**	***p*-Value**
Age	64 (54–73)	64 (56–74)	0.41
Men	268 (61%)	281 (58%)	0.42
**Severity of illness**			
APACHE IV score	72 (51–90)	70 (54–89)	0.81
Sepsis/severe sepsis	358 (82%)	394 (82%)	1.00
Septic shock	81 (18%)	88 (18%)	
SOFA score	6 (3–8)	6 (3–8)	0.59
**Acquisition of infection** ^a^			
Community-acquired	216 (49%)	229 (48%)	0.73
Hospital-acquired	111 (25%)	119 (25%)	
ICU-acquired	112 (26%)	134 (28%)	
**Presumed infection site** ^a^			
Pulmonary	276 (63%)	309 (64%)	0.15
CNS	18 (4%)	22 (5%)	
Skin and soft tissue	9 (2%)	14 (3%)	
Catheter-related	7 (2%)	8 (2%)	
Intraabdominal	63 (14%)	86 (18%)	
Urinary tract	17 (4%)	16 (3%)	
ENT	4 (1%)	1 (0.2%)	
Bloodstream	4 (1%)	2(0.4%)	
Unknown	41 (9%)	24 (5%)	
**Inflammatory parameters**			
Procalcitonin (μg/L)	2.1 (0.4–14.9)	NA	
C-reactive protein (mg/L)	230 (119–2–324)	213(121–308)	0.58
Leukocytes (10^9^/L)	14.5 (10.5–20.9)	14.8 (10.2–21.2)	0.88
Temperature (°C)	38.2 (37.5–38.9)	38.1 (37.5–38.8)	0.15

Comparison of patient characteristics at the time of randomization to procalcitonin-guide group (PCT) or control group that received standard-of-care.

Medians with (IQR) or numbers with (percentages) APACHE-IV, acute and chronic health evaluation IV score; SOFA, sequential organ failure score; CNS, central nervous system; ENT, ear nose and throat; ICU, intensive care unit.

^a^Due to rounding, percentages may not always add up to 100%.

**Table 2:** Outcomes.

	**PCT group (*n* = 439)**	**Standard-of-care group (*n* = 482)**	***p*-Value**	**Between-group absolute difference (95% CI)**
**Antibiotic use**				
Daily defined doses in first 28 d	7.9 (4.0–13.0)	9.0(5.0–17.2)	0.002	2.25 (0.30 to 4.20)
Duration of first antibiotic course	6 (4–10)	7 (5–11)	0.0001	1.03 (0.20 to 1.85)
Antibiotic-free days in first 28 d	7 (0–14)	6 (0–13)	0.05	−1.26 (−2.28 to −0.24)
Selective decontamination of the digestive tract	188 (43%)	208 (43%)	0.95	0.3% (−6.2 to 6.8)
28-day mortality	84 (19%)	114 (24%)	0.11	4.5% (−9.8 to 0.8)
ICU length of stay (days)	9 (5–18)	9 (5–18)	0.80	0.04 (−2.54–2.46)
Hospital length of stay (days)	24 (14–41)	24 (14–42)	0.76	−0.88 (−4.87 to 3.11)
**Antimicrobial resistance**				
MDR present at baseline	89 (20%)	76 (16%)	0.085	−4.5% (−9.4 to 0.4)
HRMO present at baseline	57 (13%)	50 (10%)	0.22	−2.6% (−6.7 to 1.5)
MDR or HRMO present at baseline	98 (22%)	88 (18%)	0.14	−4.1% (−9.2 to 1.0)
New MDR compared to baseline	29 (6.6%)	40 (8.3%)	0.38	1.7% (−1.6 to 5.0)
New HRMO compared to baseline	21 (4.8%)	23 (4.8%)	1.0	0.0% (−2.7 to 2.7)
New MDR or HRMO compared to baseline	39 (8.9%)	45 (9.3%)	0.82	0.5% (−3.2 to 4.2)

The PCT group had a significantly lower duration of antibiotic treatment (p = 0.0001), although this difference only amounted to 1 day. The rates of MDR and HRMO at baseline and after randomization did not differ between the two trial arms.

MDR, multidrug resistant organism; HRMO, highly resistant micro-organism according to the definition for the Netherlands (https://www.rivm.nl/documenten/wip-richtlijn-brmo; https://lci.rivm.nl/richtlijnen/brmo) as explained in the Supplementary material.

**Figure 1:**
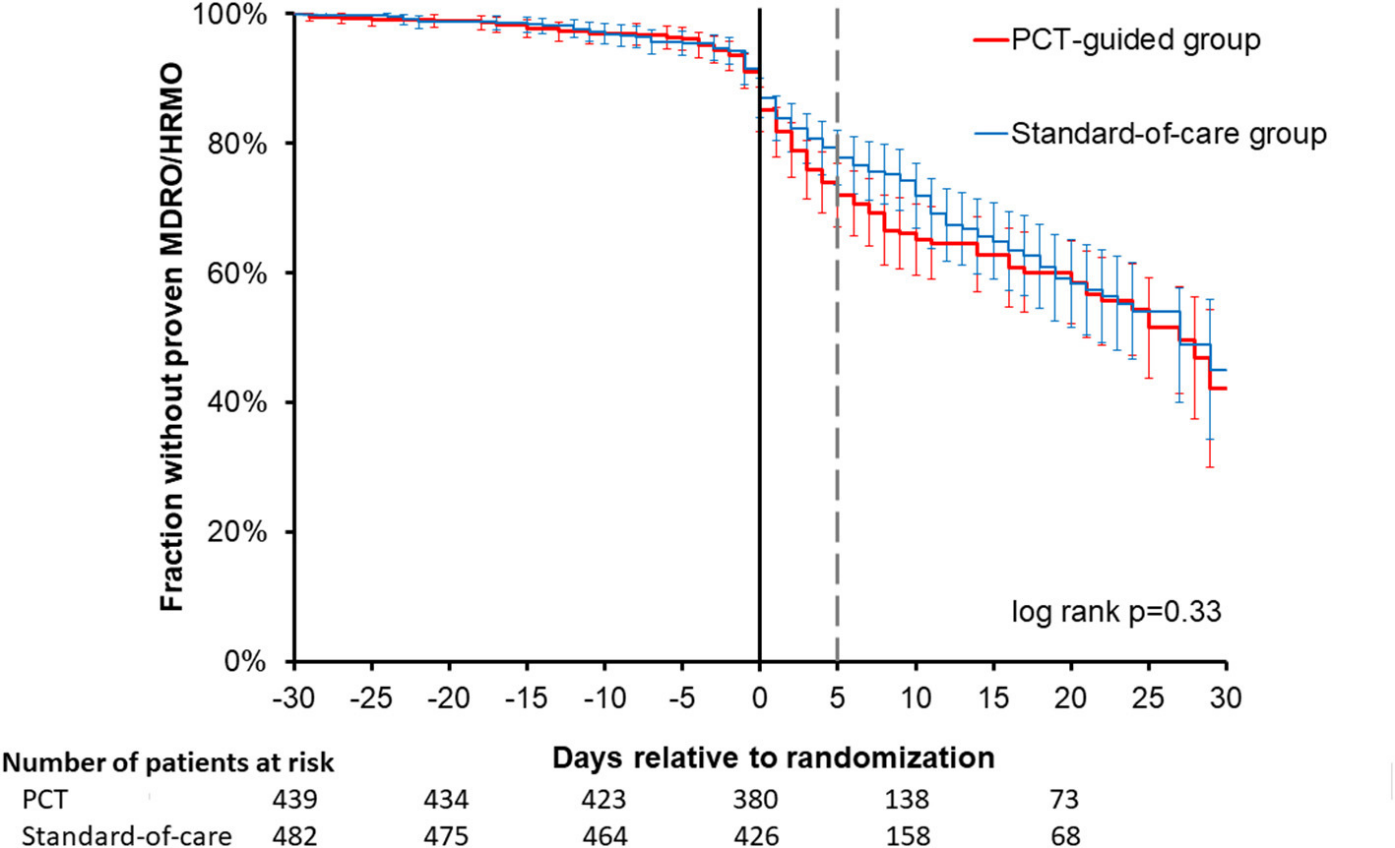
Time course of resistance development in the two trial arms. For all patients studied, the first occurrence of a multi-drug resistant (MDR) or highly resistant microorganism (HRMO) was compared between the procalcitonin (PCT) and standard-of-care groups from −30 to +30 days relative to randomization. No significant difference was observed between these two time courses. The actuarial cumulative percentages with 95% confidence intervals were plotted for the first occurrence of MDR/HRMO with the Kaplan-Meier method and compared for the PCT and control groups. The thinner, dashed vertical gray line represents the demarcation between baseline period (−30 days through +5 days) and the subsequent period (+5 days through +30 days) that we compared.

In total 8,113 cultures with 96,515 antibiotic test results were obtained. Most of the cultures were obtained around the day of randomization (Supplementary Figure 1). In total 546 isolates (7%) were non-bacterial, mainly *Candida* species. The 10 most identified bacterial isolates are shown in the Supplementary Table 5, with *Escherichia coli* (18%) being the most prominent. In only two patients *Clostridium difficile* was cultured. The five most frequently used antibiotics were ceftriaxone, ciprofloxacin, amoxicillin-clavulanate, metronidazole and cefuroxime (Supplementary Figure 2, Supplementary Table 6). Overall 1,001 (12%) of the isolates were MDR and 562 (7%) were HRMO. On a patient basis (Table 2), 22 and 18% were MDR/HRMO positive at baseline in the PCT and control groups, respectively. There were no patients with more than one unique MDR/HRMO during the study period. Subsequently, 39 (8.9%) and 45 (9.3%) of the patients became MDR/HRMO positive while they were MDR/HRMO negative at baseline (*p* = 0.82). The time course of the cumulative MDR/HRMO incidence (Figure 1) did not show a difference between the two groups (*p* = 0.33).

## Discussion

In this substudy of the SAPS trial, the baseline characteristics of the PCT and control groups were well balanced and a statistically significant difference of 1 day in ABTD was achieved. However the lower ABTD in the PCT-arm was not associated with a detectable difference in changes in MDR/HRMO incidence.

Generally, ICUs are among the heaviest consumers of antibiotics, with an estimated 70% of patients receiving antibiotics during an ICU stay (7). Various observational and before-after studies show that duration of antibiotic therapy is linked to antibiotic resistance development, both in ICU (17, 18) and non-ICU (19–21) settings. A number of randomized trials have shown that targeted interventions can safely reduce the ABTD in ICU (11, 22, 23) or non-ICU (9, 10, 12, 13) patients. Measuring readily available markers of inflammation such as C-reactive protein (CRP) or procalcitonin (PCT) can help reduce unnecessarily prolonged antibiotic prescriptions as was shown in the PRORATA (24), SAPS (8), PIRATE (13), and PROGRESS (25) trials. Although shorter antibiotic treatment is generally considered desirable, it is not beneficial under all circumstances. For example, a trial that randomized children aged 6–24 months with otitis to either 5 or 10 days of amoxicillin observed a worse outcome in the 5 days group (26). Recently the multicenter iDIAPASON trial randomized 186 patients with *Pseudomonas aeruginosa* ventilator-associated pneumonia to an ABDT of 8 or 15 days (27). Although formally non-inferiority was found, there was a trend (27) toward a better outcome in the 15 days group.

Clearly in SAPS, there was no indication that antibiotic treatment in the PCT-arm was too short, as mortality in this arm was significantly lower in the SAPS study (8), with also a trend toward lower mortality in the current substudy (Table 2). We observed a slightly smaller difference in ABTD, with a between-group absolute mean difference in ABTD of 1.03 days (Table 2) compared to 1.22 days for the original SAPS-group (8).

As expected, a clear time-dependent rise of AMR in terms of MDR/HRMO was observed after ICU admission and initiation of antibiotic treatment, as depicted in Figure 1. But the time courses were similar for groups. Although 8,113 isolates were analyzed for the 921 patients, an even larger number of cultures might have allowed the detection of more subtle differences between the two trial arms. But obtaining more isolates is not trivial, not in the least because considerable costs are associated with culturing and AMR-testing. Of note, the PIRATE trial (13) that examined CRP-guidance in limiting antibiotic treatment only reports 13 cultures for 514 randomized patients.

Studies from Belgium (15), Canada or the USA (10, 19, 22, 26, 28), China (21), France (11, 18, 23), Italy and Israel (12), Korea (29), Singapore (30), and Switzerland (13) examined the relation of ABTD with AMR. Large observational or before/after studies do indicate that prolonged ABTD increases AMR, both in (17, 18) and outside the ICU (19–21). Several meta-analyses also suggest that reduced ABTD may lead to reduced AMR (31–33). But ICU studies that randomized respectively 249 (11), 504 (13), 517 (10), and 604 (12) patients, report no significant impact on AMR. In contrast, the recent PROGRESS trial from Greece (25) does report an effect on resistance. In 261 patients randomized to PCT-guidance or standard-of-care, median ABTDs of 5 and 10 days (*p* < 0.001), respectively were achieved. Acquired resistance defined as new *C. difficile* infection of MDRO infection occurred in 7.2% and 15.3% of the patients, respectively (*p* = 0.045) (25). Possibly, the ongoing Canadian-international BALANCE trial (34) that will randomize more than 3,000 critically ill patients with a bloodstream infection to an ABTD of 7 or 14 days should also be able to detect clear differences in AMR.

Although mortality reduction was not a primary goal of the SAPS trial, in the main study with 1,546 patients we did observe better survival in the PCT arm (8), although this difference was not significant for the 921 patients from the seven centers in the current substudy (*p* = 0.11). Better adequacy of the antibiotics, more appropriate consideration of other diagnoses, decrease organ-toxicity toxicity of antibiotics as well as a type I error may all account for the observed lower mortalities.

A number of limitations of our study deserve mentioning. First, due to practical issues such as accessibility of electronic lab systems and the original design of the SAPS-trial, we were only able to obtain AMR data from seven of the original 15 participating SAPS centers, although still representing 921 patients. The resultant separation of ABTD was somewhat lower, although as indicated in Table 1, the two groups were still well matched. Second, since this sub-study of the SAPS trial combined data from different institutions with different classification systems and hospital information systems, data were not completely homogeneous. On the other hand, we believe these multicenter data well reflect routine health care in the Netherlands. Third, in the Netherlands overall antibiotic consumption is lower than many other countries (35), making it more difficult to achieve reductions larger than the 1 day reduction we achieved. Accordingly background AMR is comparatively low in the Netherlands (36). In our study, both the baseline AMR and the subsequent AMR were low when compared to many other studies that also achieved larger differences in (long) treatment durations, such as 7 vs. 14 days or 8 vs. 15 days (11, 12, 25, 26). Although randomization occurred on day 0, we somewhat arbitrarily selected day 5 as the cut-off between the baseline and subsequent periods. We chose this cut-off because of the limited number of cultures before day 0 and because divergence in ABDT as well as AMR would be expected after day 5. We did not perform subgroup analyses, since the number of patients, cultures and incidence of AMR also did not allow subgroup analyses. According to the Clinical Laboratory Standards Institute analyses should not be performed in subgroups with <30 first isolates.

With larger patient sets or larger differences in AB treatment duration or in settings with a higher background AMR, significant differences might be observed, such as in the aforementioned ongoing BALANCE trial (34). Lastly, the AMR data were obtained a decade ago—between 2009 and 2013. However we cannot conceive scientific arguments to assume that increased or decreased of AMR under PCT use would be fundamentally different in the present time, since we evaluated the relative AMR difference between PCT use and no PCT use.

In conclusion, although various types of evidence indicate that a lowered duration of antibiotic therapy protect leads to reduced subsequent AMR, our study could not demonstrate this. This may result from the small separation in antibiotic treatment duration between the two trial arms as well as the relatively low prevalence of drug-resistant organisms in the Netherlands. Future trials in large patient groups, with more marked differences in antibiotic treatment duration and in a context of higher background AMR, might be able to detect differences in subsequent AMR.

## Data availability statement

The raw data supporting the conclusions of this article will be made available by the authors, without undue reservation.

## Ethics statement

This study was approved for all centers by the Ethics Committee of the Amsterdam University Medical Center and is in compliance with the Helsinki Declaration (https://clinicaltrials.gov/ct2/show/NCT01139489). The patients/participants provided their written informed consent to participate in this study.

## Author contributions

Study concept and design: AS, MB, CL, JO, AB, DL, EJ, and MN. Acquisition of data: AS, MN, PV, RK, BL, AR, LB-R, KL, TD, MO, and AvdB. Statistical analysis: AS, MB, CL, and MN. Analysis and interpretation of data: AS, MB, and CL. Drafting of the manuscript: AS and MN. Critical revision of the manuscript for important intellectual content: AS, MB, CL, HH, DL, EJ, and MN. All authors contributed to the article and approved the submitted version.

## Footnotes

^1^https://www.rivm.nl/documenten/wip-richtlijn-brmo (accessed October 17 2022).

^2^https://lci.rivm.nl/richtlijnen/brmo (accessed October 17, 2022).
