# Supplementary material for: Impact of reduced antibiotic treatment duration on antimicrobial resistance in critically ill patients in the randomized controlled SAPS-trial
Source: Front Med (Lausanne). 2023 Feb 2;10:1080007. doi: 10.3389/fmed.2023.1080007 (PMC9932263; doi:10.3389/fmed.2023.1080007)
Supplement: Supplementary file 1 [file Data_Sheet_1.docx]

**Supplementary material file**

for “Impact of reduced antibiotic treatment duration on antimicrobial resistance in critically ill patients in the randomized controlled SAPS-trial by Shajiei A, et al.

**Definition of MDR**

Multi-drug resistant (MDR) organisms were defined as formulated by Magiorakos AP et al. in Multidrug-resistant, extensively drug-resistant and pandrug-resistant bacteria: an international expert proposal for interim standard definitions for acquired resistance. *Clin Microbiol Infect* 2012;18:268-81.

**Definition of HRMO**

For the definition of highly drug resistant micro-organisms (HRMO), the Dutch national guideline was applied (h[ttps://www.rivm.nl/documenten/wip-richtlijn-brmo](https://www.rivm.nl/documenten/wip-richtlijn-brmo)^17^, <https://lci.rivm.nl/richtlijnen/brmo>^18^, both accessed on 17.05.2022).

This guideline defines bacteria as HRMO (or BRMO “Bijzonder resistente micro-organismen”) in the context of the Netherlands. HRMOs are defined as microorganisms that are resistant to the most appropriate (i.e. first choice) antibiotics in the Netherlands, or to a combination of therapeutically important antibiotics and that can lead to spreading without additional measures, Tables 1-3, are directly based on this guideline.

**STable 1** HRMO Resistance criteria for *Enterobacteriaceae*.

| Gram-negative rods | ESBL | Fluoroquinolones | Aminoglycosides | Carbapenemase-positive |
| --- | --- | --- | --- | --- |
| *Enterobacteriaceae** | A | B | B | A |

When a single resistance according to A or at least two resistances according to B are present, the organism is considered an HRMO.

NB: From the guideline: “despite the fact that outbreaks with plasmidally AmpC-producing Enterobacteriaceae have been described in the literature, this resistance mechanism is not included in STable 1. In the Netherlands, such outbreaks have not yet been observed.“

**STable 2. Resistance criteria for non-fermenters.**

| **Gram-negative rods** | **Carbapenemase positive** | **Aminoglycosides** | **Fluoroquinolones** | **Ceftazidime** | **Pipera-cillin** | **Trimethoprim /sulfamethoxazole** |
| --- | --- | --- | --- | --- | --- | --- |
| Acinetobacter species | A | B | B* |  |  |  |
| *Stenotrophomonas maltophilia* |  |  |  |  |  | A |
| *Pseudomonas aeruginosa* | C | C | C | C | C |  |

When a single resistance according to A, or at least two resistances according to B, or at least three resistances according to C are present, the organism is considered an HRMO.

*Only ciprofloxacin and/or levofloxacin are considered, as Acinetobacter species are intrinsically resistant to norfloxacin.

**STable 3. Resistance criteria for Gram-positive cocci**

| Gram-positive cocci | Penicillin group | Vancomycin |
| --- | --- | --- |
| *Streptococcus pneumoniae* | A | A |
| *Enterococcus faecium* | B | B |

When a single resistance according to A or at least two resistances according to B are present, the organism is considered an HRMO.

**STable 4 Materials used for culture**

| **Material** | **N (%)** |
| --- | --- |
| wound/pus | 946 (26%) |
| sputum | 871 (24%) |
| rectum/faeces | 498 (14%) |
| blood | 408 (11%) |
| throat | 297 (8%) |
| urine | 231 (6%) |
| BAL | 132 (4%) |
| intravascular catheter | 108 (3%) |
| tissue | 65 (2%) |
| misc | 97 (3%) |

**Legend:** For only 3653 (45%) of the cultures, the source could be retrieved.

**STable 5: Ten most frequently cultured bacteria.**

| **Organism** | **N (%)** |
| --- | --- |
| *E coli* | 1499 (18%) |
| *S aureus* | 806 (10%) |
| *P aeruginosa* | 774 (10%) |
| *Coagulase negative Staphylococcus* | 562 (7%) |
| *E cloacae* | 459 (6%) |
| *K pneumoniae* | 436 (5%) |
| *E faecium* | 300 (4%) |
| *K oxytoca* | 210 (3%) |
| *E faecalis* | 207 (3%) |
| *S marcescens* | 207 (3%) |

**SFigure 1 Time distribution of isolates**


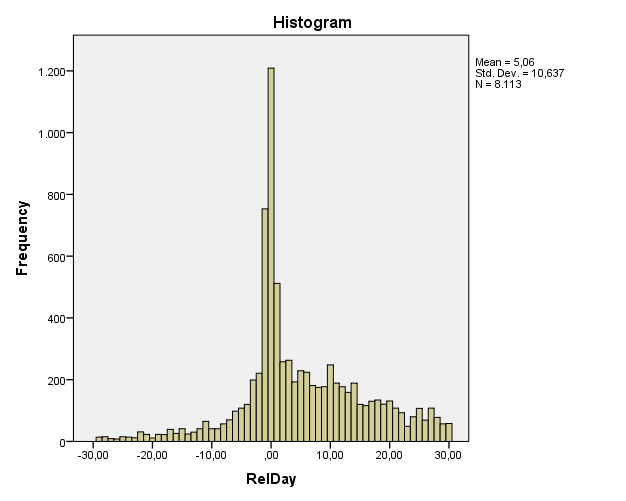


**Legend:** Distribution of time of the 8113 isolates that were analyzed. **RelDay** denotes the day the isolates were obtained relative to randomization.

**SFigure 2**

**Time distribution of antimicrobial drugs administered.**

**Legend:** The five most frequently used initial antibiotics were ceftriaxone (CRO), ciprofloxacin (CIP), amoxicillin-clavulanate (AMC), metronidazole (MET) and cefuroxime (CXM). Note that the total number exceeds the number of patients (N=921) because of combination treatments. For legends of all 3-letter abbreviations used, see **STable 6** below.

**STable 6**

**Time distribution of antimicrobial drugs administered.**

**Legend:** The numbers in this table correspond to the numbers displayed in **SFigure 2**.
